# The use and diagnostic yield of radiology in subjects with longstanding musculoskeletal pain – an eight year follow up

**DOI:** 10.1186/1471-2474-6-53

**Published:** 2005-11-03

**Authors:** Hans Lindgren, Stefan Bergman

**Affiliations:** 1 Helsingborg County Hospital, Helsingborg, Sweden; 2 Spenshult Hospital for Rheumatic Diseases, Halmstad, Sweden

## Abstract

**Background:**

Longstanding musculoskeletal pain is common in the general population and associated with frequent use of health care. Plain radiography is a common diagnostic approach in these patients despite knowledge that the use in the investigation of musculoskeletal pain is associated with low diagnostic yield, substantial costs and high radiation exposure. The aim of this study was to assess the use of diagnostic imaging and the proportion of pathological findings with regard to duration and distribution of pain in a cohort from the general population.

**Methods:**

An eight-year longitudinal study based on questionnaires at three occasions and medical records on radiological examinations done in medical care. Thirty subjects were selected from an established population based cohort of 2425 subjects that in 1995 answered a postal survey on pain experience. At baseline there were ten subjects from each of three pain groups; No chronic pain, Chronic regional pain, and Chronic widespread pain (CWP). Those who presented with CWP at two or all three occasions were considered to have a longstanding or re-occurring CWP. In total the thirty subjects underwent 102 radiological examinations during the eight year follow up.

**Results:**

There was a non-significant (p = 0.10) finding indicating that subjects with chronic pain at baseline (regional or widespread) were examined three times more often than those with no chronic pain. When the indication for the examination was pain, there was a low proportion of positive findings in subjects with longstanding CWP, compared to all others (5.3% vs 28.9%; p = 0.045). On the other hand, in examinations on other indications than pain the proportion of positive findings was high in the CWP group (62.5% vs 14.8%; p = 0.001).

**Conclusion:**

Radiological examinations had a low diagnostic yield in evaluation of pain in subjects with longstanding/reoccurring CWP. These subjects had on the other hand more often positive findings when examined on other indications than pain. This may indicate that subjects with longstanding/reoccurring CWP are more prone to other diseases. It is a challenge for caregivers, often primary care physicians, to use radiological examinations to the best for their patients.

## Background

Several studies from Western Europe and the USA have shown that chronic (most often defined as a duration of three months or more) musculoskeletal pain is common in the general population, with a prevalence (point or one year period) of 30–50% [1-3]. With regard to distribution of musculoskeletal pain in the body, a distinction is often made in epidemiological studies between chronic regional pain (CRP) and chronic widespread pain (CWP) [1,3,4]. In postal surveys approximately 11% of subjects in a general population report CWP [1-3]. Chronic musculoskeletal pain, and foremost CWP, has been shown to have other background factors than acute and more localized pain [5]. CWP is to a higher degree associated to adverse psychosocial and sociodemographic factors. Besides being common in the population, musculoskeletal pain is also associated with frequent use of health care. That is especially true for subjects with low-back and widespread pain [6].

Plain radiography is a common diagnostic approach in these patients despite knowledge that the use in the investigation of musculoskeletal pain could be associated with low diagnostic yield, substantial costs and high radiation exposure [7,8]. This is well studied in plain radiography for low back pain, were studies have shown that 25–50% may be unnecessary according to clinical recommendations [9,10]. There are internationally recognised guidelines concerning the use of radiography in the management of patients with low-back pain [11,12]. These guidelines have not substantially changed the pattern of doctors' referrals [13]. Patient expectations can also be an explanation. It has for example been reported that the majority (72%) of patients referred to radiological examination because of low back pain rate this as very important [14].

The use of radiography of the lumbar spine in primary care patients with low back pain has not only implications on diagnostic yield and costs, but has also been reported to be associated with a worse patient outcome and an increased doctors workload [15]. Furthermore the radiation exposure could be high, especially in radiography of the lumbar spine, where the gonadal dose for one investigation in women is equivalent to a daily chest radiograph over six years [16].

The aim of this study was to assess if subjects with chronic musculoskeletal pain (CRP and CWP) were submitted to more radiological investigations than those with no chronic pain. A second aim was to study the proportion of pathological findings with regard to duration and distribution (no chronic pain, CRP, and CWP), and the indication for referral to the examination (pain or other causes).

## Methods

A sample of 30 subjects was derived from a cohort of 2755 subjects that in 1995 responded to a postal survey on experience of pain. This survey included initially a representative sample of 3928 subjects aged 20–74 years, living in two municipalities on the west coast of Sweden. According to pain history and pain distribution on a pain drawing the respondents were categorised into three groups: No chronic pain (NCP), Chronic regional pain (CRP), and Chronic widespread pain (CWP). Chronic pain was defined as pain more than three months during the last year. Widespread pain was defined according to ACR 1990 criteria for fibromyalgia. Details can be found in previous work [1]. Ten subjects from each of the three groups (NCP, CRP and CWP) were randomly selected and included in this study.

From the selection of 30 subjects, 28 had also answered follow up postal surveys in 1998 and 2003. These 28 subjects were for sub-analyses further divided into two groups; (1) those belonging to the CWP-group in at least two of the postal surveys (n = 5), and (2) all others (n = 23).

The radiology records between 1994 and 2002 were scrutinised and all radiographs were double-checked by the authors. The results were in total agreement with the original readers of the radiology examinations. A total of 102 examinations were included in the study. It was also noted if the referral was due to "pain" or "other cause", such as suspected infection or malignancy, and if the examination resulted in a positive finding or not.

Non-parametrical statistical tests in the statistical package SPSS 12.0 were used for the analyses. Mann-Whitney U-test was used when comparing number of radiological examinations for each individual in the different pain-groups. Chi-squared test was used for group comparisons. The study was approved by the Ethics Research Committee, Faculty of Medicine, University of Lund, Sweden.

## Results

Out of the total sample of 30 subjects, there were 14 who had been examined by radiology, and together they had been submitted to 102 examinations. In 61% of the cases the referral was from a general practitioner and in 88% the examination was done in outpatient care. A majority of the examinations (66%) were scheduled and not acute. An overview of the examinations and their outcome show positive findings in 25.5% of the cases (Table 1).

**Table 1:** Radiological examinations and positive findings. The number of different radiological examinations from 1994 to 2002, and the number of positive findings.

Examination	Number of examinations	Positive findings (%)
Peripheral skeleton	30	5 (16.7)
Axial skeleton	18	5 (27.8)
Pulmonary	24	10 (41.7)
Mammography	9	0 (0)
CT/MR	7	2 (28.6)
Other	14	4 (28.6)

Total	102	26 (25.5)

The results from the examinations were further analysed with regard to pain group (NCP, CRP or CWP) belonging in 1995, and information regarding the indication for referral (pain or other cause). The mean age varied from 36 in the NCP-group, over 47 in the CRP-group, to 53 in the CWP-group. There was two-times more women than men in the CWP-group, but no such difference in the NCP- or CRP-groups. There was a non-significant (p = 0.10) finding indicating that subjects with chronic pain (regional or widespread) were examined more often than those with no chronic pain (Table 2). The outcome of the radiological examinations (positive or negative findings) varied nearly significantly (p = 0.08) with the indication for the examination ("pain" or "other cause"), with overall less positive findings in examinations done in the pain group. Most prominent result is the high proportion of positive findings (62.5%; p = 0.013) in the CWP-group examined on other indication than pain (Table 3).

**Table 2:** Number of examinations with respect to pain distribution. The number of radiological examinations in each subject from 1994 to 2002, for each of the three paingroups; chronic widespread pain (CWP), chronic regional pain (CRP, and those with no chronic pain (NCP).

Pain group	Number of subjects	Number of examinations	Exam's/subject
CWP	10	48	4.8
CRP	10	40	4.0
NCP	10	14	1.4

Total	30	102	3.4

**Table 3:** Positive findings and the indication for radiological examination. The number of positive findings on radiological examination with regard to the indication for the examination (pain or other cause).

	Examinations due to pain		Examinations due to other causes	
Pain group	Number of examinations	Number of positive findings (%)	Number of examinations	Number of positive findings (%)
CWP	32	8 (25.0)	16	10 (62.5)
CRP	22	4 (18.2)	18	3 (16.7)
NCP	5	0 (0.0)	9	1 (11.1)

Total	59	12 (20,3)	43	14 (32,6)

CWP = chronic widespread pain, CRP = chronic regional pain, NCP = no chronic pain

The radiological examinations were done during an eight-year period. It was decided to also take changes in pain group belonging under the time period into account in the further analyses. The results from the analysis above concerning the outcome in those with CWP at baseline motivated that subjects with longstanding/reoccurring CWP (two or more occasions during the eight year period) were compared to all others. The two groups did not differ in mean age, but the group with longstanding/reoccurring CWP had an over-representation of women. There was a non-significant finding (p = 0.24) that those with longstanding/reoccurring CWP were examined more than twice as often than all others (Table 4). If the indication for the examination was pain, there was a low proportion of positive findings in those with longstanding/reoccurring CWP compared to all others (5.3% vs. 28.9%; p = 0.045; Table 5). On the other hand, if the indication for the examination was other than pain, the proportion of positive findings in subjects with longstanding/reoccurring CWP was as high as 62,5%, compared to 14.8% amongst all others (p = 0.001; Table 5).

**Table 4:** Number of examinations and longstanding/reoccurring widespread pain. The number of radiological examinations in each subject from 1994 to 2002 for those with longstanding/reoccurring (reported in at least two of the three surveys) chronic widespread pain (CWP) compared to all others.

Pain group	Number of subjects	Number of examinations	Exam/subjects
Two times CWP	5	35	7.0
All others	23	65	2.8

Total	28	100	3.6

**Table 5:** Positive findings and the indication for radiological examination. The number of positive findings on radiological examination with regard to the indication for the examination (pain or other cause), for those with longstanding/reoccurring (reported in at least two of the three surveys) chronic widespread pain (CWP) compared to all others.

	Examinations due to pain		Examinations due to other causes	
Pain group	Number of examinations	Number of positive findings (%)	Number of examinations	Number of positive findings (%)
Two times CWP	19	1 (5.3)	16	10 (62.5)
All others	38	11 (28.9)	27	4 (14.8)

Total	57	12 (21.1)	43	14 (32.6)

## Discussion

A main finding in this study was that subjects with chronic regional or widespread pain were more frequently submitted to radiological examinations than those with no chronic pain. The diagnostic yield was low if the indication for the examination was pain. On the other hand there was a high proportion of positive findings if the indication for the examination was other than pain. This was especially true for subjects with longstanding/reoccurring CWP during the eight-year follow up period.

During scrutinising the radiographs and when reviewing the literature it was found questionable how to define a significant positive finding with regard to degenerative changes in the spine. In the analyses it was decided to count five cases of degenerative spinal changes as negative results. Such findings are very common in lumbar spine radiology. The prevalence of degenerative changes in the lumbar spine rises with age with an occurrence of as much as 71% in the age group 65–74 years. Their relation to pain has been considered speculative, and the therapeutic consequences of these findings are minor [13].

We found no cases with disc herniation, by many clinicians considered a major positive finding, though this has been reported in as much as 20–70 % in pain free populations [17-19]. This was not surprising since plain radiography is considered relatively insensitive for disc herniation or more serious spinal conditions [20]. Magnetic resonance imaging (MRI) has been proposed as an alternative investigation for patients with low back pain [21]. MRI has however not been found to give a better outcome to primary care patients with low back pain and may increase the cost of care [22]. It has been stated that the majority of patients with low back pain should be assessed clinically and that radiological imaging seldom is required. [23].

Subjects with longstanding/reoccurring CWP have often a multifactorial background to their pain problem. The patophysiology in the musculoskeletal system could be of minor importance when pain is maintained in an interaction between neurophysiological processes and psychosocial factors [5]. It is thus understandable that the diagnostic yield is low when radiological examinations are done to find the cause of pain in the musculoskeletal structures.

There were more positive findings in the CWP group when radiological examinations were done on other indications than pain. This can be an expression of a higher incidence of other diseases amongst subjects with CWP. One important clinical implication of this is, that if an individual with chronic widespread pain seeks his/her doctor for a reason other than pain – there is good reason for alert and to go further on with diagnostic tests if necessary.

It has been reported that inappropriately referred patients tended to rate their radiography referrals more important than appropriately referred patients [14]. The patient's view could thus be a barrier to a more justified use of radiography. This is a challenge to the caregivers but a more appropriate use of radiography could be supported by guidelines [15].

Some argue that the usage of radiographs is justified to rule out serious disease and to reassure the patient. However in low back pain the prevalence of possible serious conditions (fracture, infection or tumour) is very low, which implies radiation exposure in many patients with no significant lesion [13]. The radiation dose from lumbar radiographs in a given patient is 40 times the dose received from chest radiography, with gonadal doses for women equivalent to daily chest radiograph over six years [16]. The risk from any investigation must be justified by being less harmful than the potential risk when neglecting to do it. In radiological investigations, due to the risk of radiation-induced malignancy, the radiation exposures must be justified and optimised [24].

The strength of the present study lies in that it is based on a cohort from the general population living in a well defined area, that have been followed over an eight year period. Pain experience have been recorded on three occasions during this eight year period, and there is only one hospital performing the radiological examinations in the area. The major drawback is the relatively small material that also gives power problems in some of the analyses. The age distribution between groups and the higher proportion of women with CWP is expected from previous studies [1], but could introduce a bias in the first analysis (Tables 2 and 3) with a presumed higher referral rate with age. The results were however consistent in the second analysis (Tables 4 and 5), where there was no difference in age between the two studied groups. The external validity is strengthened by the design with a sample from the general population but weakened by the small size of the study. The results must thus be interpreted by this in mind.

## Conclusion

Radiological examinations had a low diagnostic yield in evaluation of pain in subjects with longstanding/reoccurring CWP. These subjects had on the other hand often positive findings when examined on other indications than pain. This may indicate that subjects with longstanding/reoccurring CWP are more prone to other diseases. It is a challenge for caregivers, often primary care physicians, to use radiological examinations to the best for their patients.

## Competing interests

The author(s) declare that they have no competing interests.

## Authors' contributions

SB was responsible for the Epipain project that established the pain cohorts. Both authors were equally involved in all aspects of the present study. Both authors read and approved the final manuscript.

## Pre-publication history

The pre-publication history for this paper can be accessed here:


